# WHO European Childhood Obesity Surveillance Initiative: associations between sleep duration, screen time and food consumption frequencies

**DOI:** 10.1186/s12889-015-1793-3

**Published:** 2015-04-30

**Authors:** Claudia Börnhorst, Trudy MA Wijnhoven, Marie Kunešová, Agneta Yngve, Ana I Rito, Lauren Lissner, Vesselka Duleva, Ausra Petrauskiene, João Breda

**Affiliations:** Department of Biometry and Data Management, Leibniz Institute for Prevention Research and Epidemiology – BIPS GmbH, Achterstrasse 30, 28359 Bremen, Germany; Division of Noncommunicable Diseases and Promoting Health through the Life-Course, WHO Regional Office for Europe, UN City, Marmorvej 51, DK-2100 Copenhagen Ø, Denmark; Obesity Management Centre, Institute of Endocrinology, Narodni 8, 11694 Prague 1, Czech Republic; School of Hospitality, Culinary Arts and Meal Science, Örebro University, Campus Grythyttan, P.O. Box 1, SE 712 02 Grythyttan, Sweden; National Health Institute Doutor Ricardo Jorge, Av. Padre Cruz, 1649-016 Lisbon, Portugal; Section for Epidemiology and Social medicine (EPSO), Department of Public Health and Community Medicine, University of Gothenburg, P.O. Box 454, SE 405 30 Gothenburg, Sweden; Department of Food and Nutrition, National Center of Public Health and Analyses, 15 Akad. Ivan Evstatiev Geshov Blvd, 1431 Sofia, Bulgaria; Department of Preventive Medicine, Lithuanian University of Health Sciences, Eiveniu str. 4, 50009 Kaunas, Lithuania

**Keywords:** Sleep, Screen time, Food frequency, TV viewing, Computer use, Childhood overweight, Cross-sectional study, Snacks, Europe

## Abstract

**Background:**

Both sleep duration and screen time have been suggested to affect children’s diet, although in different directions and presumably through different pathways. The present cross-sectional study aimed to simultaneously investigate the associations between sleep duration, screen time and food consumption frequencies in children.

**Methods:**

The analysis was based on 10 453 children aged 6–9 years from five European countries that participated in the World Health Organization European Childhood Obesity Surveillance Initiative. Logistic multilevel models were used to assess associations of parent-reported screen time as well as sleep duration (exposure variables) with consumption frequencies of 16 food items (outcome variables). All models were adjusted for age, sex, outdoor play time, maximum educational level of parents and sleep duration or screen time, depending on the exposure under investigation.

**Results:**

One additional hour of screen time was associated with increased consumption frequencies of ‘*soft drinks containing sugar*’ (1.28 [1.19;1.39]; odds ratio and 99% confidence interval), ‘*diet/light soft drinks*’ (1.21 [1.14;1.29]), ‘*flavoured milk*’ (1.18 [1.08;1.28]), ‘*candy bars or chocolate*’ (1.31 [1.22;1.40]), ‘*biscuits, cakes, doughnuts or pies*’ (1.22 [1.14;1.30]), ‘*potato chips (crisps), corn chips, popcorn or peanuts*’ (1.32 [1.20;1.45]), ‘*pizza, French fries (chips), hamburgers*’(1.30 [1.18;1.43]) and with a reduced consumption frequency of ‘*vegetables (excluding potatoes)*’ (0.89 [0.83;0.95]) and *‘fresh fruits’* (0.91 [0.86;0.97]). Conversely, one additional hour of sleep duration was found to be associated with increased consumption frequencies of ‘*fresh fruits*’ (1.11 [1.04;1.18]) and ‘*vegetables (excluding potatoes)*’ (1.14 [1.07;1.23]).

**Conclusion:**

The results suggest a potential relation between high screen time exposure and increased consumption frequencies of foods high in fat, free sugar or salt whereas long sleep duration may favourably be related to children’s food choices. Both screen time and sleep duration are modifiable behaviours that may be tackled in childhood obesity prevention efforts.

**Electronic supplementary material:**

The online version of this article (doi:10.1186/s12889-015-1793-3) contains supplementary material, which is available to authorized users.

## Background

The rising prevalence of overweight and obesity in children during the last decades is alarming. Childhood obesity is likely to track into adulthood and may result in metabolic, musculoskeletal or cardiovascular diseases and increase cancer risk [1-3]. One main factor suggested for the emerging obesity epidemic relates to the changes in lifestyles. Today children may spend more time with electronic media than with any single activity other than sleeping [4]. Often televisions (TV), personal computers (PC), smart phones or game consoles are used concurrently – all of which encourage sedentary behaviour. In addition, the availability of electronic media in children’s bedrooms has increased dramatically, which was found to be a significant determinant of total screen time [5]. In this context, positive associations between having a TV in a child’s bedroom and overweight/obesity were also reported [6]. To date, the pathway of the effect of screen time on overweight/obesity is not completely understood. The displacement of more active pursuits by spending time with electronic media has been suggested to lead to obesity, but this hypothesis is not fully supported by research evidence [7-9]. Screen time may further affect weight status through its impact on energy intake [10-12]. As a potential direct mechanism, Bellissimo et al. suggested TV viewing while eating to alter energy intake by delaying satiation and reducing satiety signals from previously consumed foods [11]. Another discussed pathway refers to the exposure to advertising of foods high in saturated fats, *trans*-fatty acids, free sugars or salt, which may increase children’s requests for those products [13]. Early socialization of children to associate TV time with snacking of unhealthy foods exemplified through parents may further explain the association of TV time with excess energy intake [14]. Consequently, screen time may operate through its effect on energy intake in addition to the displacement of high-energy-expending activities by sedentary activities [10,15].

Moreover, TV viewing and PC use were both shown to be associated with shorter sleep duration [5]. In a systematic review of world literature, consistent rapid declines in the sleep duration of children and adolescents over the last 100 years have been reported [16], which may partially be attributed to increases in total screen time. Short sleep duration is another known risk factor for obesity [17,18]. Also the exact mechanism through which sleep duration affects overweight/obesity is not yet clear. Apart from behavioural hypotheses like the “less sleep – more time to eat” one [19,20], several endocrinological mechanisms have been discussed to explain this association including pathways via insulin, cortisol, ghrelin and leptin [21]. For example, the appetite-lowering hormone leptin was reported to be less secreted in periods of short sleep duration whereas ghrelin, an appetite-stimulating hormone, was shown to be elevated. These sleep-induced hormonal changes may lead to alterations in appetite, hunger and food consumption and may hence promote overweight/obesity through its effect on diet [22-25].

Summing up, screen time and sleep duration seem to be interrelated and both have been suggested to affect children’s eating behaviours, although in different directions and presumably through different pathways [26,27]. Therefore, the present study aims to simultaneously investigate the effects of screen time and sleep duration on children’s food consumptions. Both of these exposures are modifiable behaviours, and thus the results may be of public health interest, in particular for policy-makers as they may provide insights to prevent the excessive weight gain particularly in young children.

## Methods

### World Health Organization European Childhood Obesity Surveillance Initiative

In 2006, the World Health Organization (WHO) Regional Office for Europe initiated with 13 Member States the WHO European Childhood Obesity Surveillance Initiative (COSI). These 13 countries were: Belgium (Flemish region only), Bulgaria, Cyprus, Czech Republic, Ireland, Italy, Latvia, Lithuania, Malta, Norway, Portugal, Slovenia and Sweden. COSI aims to measure trends in overweight and obesity in children aged 6.0–9.9 years, in order to monitor the progress of the epidemic and to permit intercountry comparisons. The first COSI data collection round took place from September 2007 to December 2008. The actual measurement period within this data collection round, however, could vary across the countries [28]. Besides the mandatory measurements of the children’s weight and height, the COSI protocol also includes the option to gather information, including simple indicators of the children’s dietary intake and physical activity/inactivity patterns as well as on parental education [29]. This was done through a so-called COSI family form of which its questions were partly based on the questionnaire used in the 2001/2002 round of the Health Behaviour in School-aged Children study [30]. In the first COSI round five countries (Bulgaria, Czech Republic, Lithuania, Portugal, and Sweden) chose for this option. The present paper is based on children from these five countries with information on weight, height and a filled-out family form.

The COSI protocol is in accordance with the international ethical guidelines for biomedical research involving human subjects [31]. All procedures were also approved by local ethical committees: Bulgaria: the Medical Ethical Committee of the former National Centre of Public Health Protection; Czech Republic: the Ethical Committee of the Institute of Endocrinology; Lithuania: Lithuanian Bioethics Committee; Portugal: Portuguese Data Protection Authority; Sweden: regional ethics review boards in Gothenburg and Stockholm. Parents were fully informed about all study procedures, and their written informed consent was obtained using either an active or passive approach. Children’s consent was always obtained prior to the anthropometric measurements [28,29].

Taking into account the local arrangements and available budget, countries chose the most appropriate professionals to measure the children’s weight and height (e.g. physical education teachers, nationally or regionally based health professionals). The family record form was either given directly to the parents, sent home with the child or mailed to the home and was generally completed jointly by the children’s caregivers and their child. A more detailed description of the implementation characteristics of the first COSI round can be found elsewhere [29].

### Sampling of children

Nationally representative samples of children were drawn whereby two-stage cluster sampling was applied using the primary school as the primary sampling unit and school classes as the secondary sampling unit. Primary schools were selected randomly from the list of all primary schools centrally available in each country through the ministry of education or at the national school registry. As an exception, paediatric clinics formed the primary sampling units for the Czech Republic which were randomly selected from the national list of primary care paediatricians. COSI targets 6-, 7-, 8- and 9-year-old children whereby countries could choose one or more of these four age groups. Four of the five countries that are subject of the present paper’s research (Bulgaria, the Czech Republic, Lithuania and Portugal) targeted one age group (7-year-olds) and Sweden targeted two age groups (7- and 8-year-olds). If all children of the specifically targeted age group were in the same grade, then one class per school was drawn within a grade level. If the specifically targeted age group was spread across grades, however, all grades where children from this age group were present could be sampled. All children registered in the sampled classes with parental informed consent and who were present on the survey day (Bulgaria: n = 3914, Czech Republic: n = 1695, Lithuania: n = 4955, Portugal: n = 3592, and Sweden: n = 5338) were approached for the anthropometric measurements and were given the family record form. Further details about the sampling characteristics have been described elsewhere [28,29].

### Exposure variables

#### Sleep duration

Sleep duration was reported as *‘hours ___ minutes ___ of usual amount of sleep per day’*. Parents were instructed to consider nocturnal and daytime sleep (e.g. naps) of their child as well as weekdays and weekends when answering this question. For the descriptive statistics, sleep duration was dichotomized into the categories “short” (< population mean) and “long” (≥ population mean) based on the average sleep duration of the whole study population in this analysis (9.7 hours/day).

#### Screen time

Information on screen time was assessed querying the number of hours per day the child usually ‘*a) spends watching TV (including videos)’* or ‘*b) spends using a computer (PC) for playing games (other than homework)’* at home or somewhere else, separately for weekdays and weekend days. Five answer categories were indicated on the form and, in the analysis, numeric values were assigned to these five categories to convert the variables to a numerical scale (“never” = 0; “<1 hour per day” = 0.5; “1 hour per day” = 1; “2 hours per day” = 2; “3 or more hours per day” = 3). Usual total screen time per day was calculated as the sum of TV and PC time weighing weekday (5/7) and weekend viewing hours (2/7) accordingly, i.e.

$$ \begin{array}{l} Usual\  total\  screen\  time\  per\  day\ (hours) = 5/7*\left( reported\  hours\  of\ PC + TV\  time\  at\  weekdays\right) + 2/7*\hfill \\ {}\left( reported\  hours\  of\ PC + TV\  time\  at\  weekend\  day s\right).\hfill \end{array} $$

For the descriptive statistics, screen time exposure was dichotomized into the categories “low” (<2 hours per day) and “high” (≥2 hours per day) based on the recommendation of the American Academy of Pediatrics to limit total media time to no more than 1 to 2 hours per day [32].

###  Outcome variables

#### Food consumption frequencies

Information on food consumption frequencies was obtained through a short food frequency questionnaire (FFQ) which had been adapted from the 2001/2002 questionnaire of the Health Behaviour in School-aged Children study [30] that has been validated among adolescents [33]. The FFQ queried consumption frequencies over a typical week for 16 food items (in days per week): ‘*fresh fruits*’ , ‘*vegetables (excluding potatoes)*’ , ‘*100% fruit juice*’ , ‘*soft drinks containing sugar*’ , ‘*diet/light soft drinks*’ , ‘*low-fat/semi-skimmed milk*’ , ‘*whole-fat milk*’ , ‘*flavoured milk*’ , ‘*cheese*’ , ‘*yoghurt, milk pudding, cream cheese/quark or other diary products*’ , ‘*meat*’ , ‘*fish*’ , ‘*potato chips (crisps), corn chips, popcorn or peanuts*’ , ‘*candy bars or chocolate*’ , ‘*biscuits, cakes, doughnuts or pies*’ , ‘*pizza, French fries (chips), hamburgers, sausages or meat pies*’). No information on portion sizes was assessed. Four answer categories were indicated on the form and, in the analysis, numeric values were assigned to these four categories to convert the variables to a numerical scale, allowing the calculation of average consumption frequencies in days per week (“every day” = 7; “4–6 days” = 5; “1–3 days” = 2; “never” = 0). For the logistic models, binary variables were constructed by combining the first/second as well as the third/fourth category (≥4 days/week vs <4 days/week) of every food item into one category each. Other categorizations were chosen for ‘*fresh fruits*’ and ‘*vegetables (excluding potatoes)*’ , which are considered as food items that are consumed daily (every day vs <7 days/week) as well as for ‘*soft drinks containing sugar*’ and ‘*diet/light soft drinks*’ , which are considered as episodically-consumed food items (≥1 day/week vs. never).

### Covariates

#### Age-adjusted body mass index z-scores (BMI/A z-scores)

The children’s weight and height were measured according to WHO standardized techniques [34] as well as information on their age and sex noted by trained fieldworkers. Children removed their shoes and socks as well as heavy clothing. Body weight was measured to the nearest 0.1 kg with portable digital (mainly manufacturer-calibrated) scales, and body height was measured standing upright to the nearest 0.1 cm with portable stadiometers. Body weight was adjusted for the weight of the clothes worn. BMI was calculated based on the formula [weight (kg)]/[height (m)]^2^. The 2007 WHO recommended sex- and age-specific cut-offs for school-aged children and adolescents were used to compute BMI/A z-scores [35]. Thinness was defined as the proportion of children with a BMI/A value below −2 z-scores, overweight and obesity were defined as the proportion of children with a BMI/A value above +1 z-score and above +2 z-scores, respectively.

#### Outdoor play time

The number of hours the child usually played outside (at home or somewhere else) was assessed for both weekdays and weekend days. Five answer categories were indicated on the form and, in the analysis, numeric values were assigned to these five categories to convert the variables to a numerical scale (“never” = 0; “<1 hour per day” = 0.5; “1 hour per day” = 1; “2 hours per day” = 2; “3 or more hours per day” = 3). Analogous to the calculation of usual screen time, usual outdoor play time was calculated weighing weekday (5/7) and weekend hours (2/7) accordingly.

#### Educational level of parents

Information on the highest educational level completed by both parents was assessed based on the following four answer categories: “primary school”, “secondary school”, “undergraduate/bachelor’s degree”, “master’s degree or higher”. The maximum educational level of both parents was taken and, based on this, a binary variable (first/second versus third/fourth category) was constructed that was used for adjustment in later multivariable models.

### Statistical analyses

All country datasets were reviewed in a standard manner for inconsistencies and completeness at the WHO Regional Office for Europe before being merged for the intercountry analyses. The initial dataset included 15 692 children with complete information on age, sex, weight and height and a filled-out family record form. Children with biologically implausible BMI/A z-scores below −5 or above +5 z-scores relative to the 2007 WHO growth reference median (n = 31) were excluded from the analysis as well as children who were younger than 6 years (n = 5) or older than 9 years (n = 13) and children with a missing value for any of the exposure variables, outcome variables or covariates (Bulgaria: n = 1020, Czech Republic: n = 436, Lithuania: n = 1319, Portugal: n = 1242, Sweden: n = 1193, total: n = 5210). The remaining dataset consisted of 10 453 children from five countries. No significant differences in terms of study subjects’ characteristics (age, sex and BMI category) were found between the sample included in the present analyses and the initial sample.

Multivariable logistic multilevel models were used to assess associations between screen time as well as sleep duration (exposure variables) and the consumption frequencies of the 16 food items (outcome variables). In a first step, only screen time (but not sleep duration) was included in the logistic multilevel models. Sleep duration was added to the models in a subsequent step to investigate how the effect estimate of screen time changes by inclusion of sleep duration, i.e. to check for a mediation effect. In all cases, the effect estimates of screen time on the single food items remained nearly unchanged when adding sleep duration to the models. For this reason, in the final analysis, screen time (hours/day) and sleep duration (hours/day) were simultaneously included in the 16 models for the single food items, i.e. models for screen time exposure were adjusted for sleep duration and vice versa. All models were further adjusted for age (continuously: years), sex (binary; ref: boys), outdoor play time (continuously: hours/day) and maximum parental educational level (binary; ref: primary/secondary school) and included random effects for the country and for the primary sampling units (schools in Bulgaria, Lithuania, Portugal, Sweden; paediatric clinics in the Czech Republic) to account for the clustered study design. In a subsequent step, all models were additionally adjusted for BMI/A z-scores of the children (continuously). P-values below 0.01 were considered as statistically significant to account for multiple testing. All analyses were conducted using the statistical analysis software SAS (version 9.3; SAS Institute, Cary, NC, USA).

## Results

A description of the study sample in terms of age, sex, country, BMI category and maximum educational level of parents is given in Table 1. Both sexes were almost equally represented (boys: 5290 (50.6%); girls 5163 (49.4%)) and most children were aged between 7.0 and 8.9 years (n = 8901 (85.1%)). In the total study group, 26.9% of the children were classified as overweight or obese, but prevalence estimates differed by country (20.4 − 37.4%).

**Table 1:** **Study participants characteristics (total study group and by BMI category)**

**Characteristics**	**Total study group**	Thinness and normal weight*	Overweight (including obesity)*
	**n (%)**	**n (%)**	**n (%)**
**Age group (years)**			
6.0–6.9	1098 (10.5)	803 (73.1)	295 (26.9)
7.0–7.9	5857 (56.0)	4269 (72.9)	1588 (27.1)
8.0–8.9	3044 (29.1)	2216 (72.8)	828 (27.2)
9.0–9.9	454 (4.3)	353 (77.8)	101 (22.3)
**Sex**			
Boys	5290 (50.6)	3788 (71.6)	1502 (28.4)
Girls	5163 (49.4)	3853 (74.6)	1310 (25.4)
**Country**			
Bulgaria	2251 (21.5)	1592 (70.7)	659 (29.3)
Czech Republic	1206 (11.5)	960 (79.6)	246 (20.4)
Lithuania	2767 (26.5)	2081 (75.2)	686 (24.8)
Portugal	1788 (17.1)	1119 (62.6)	669 (37.4)
Sweden	2441 (23.4)	1889 (77.4)	552 (22.6)
**Maximum educational level of parents**			
Primary school	804 (7.7)	589 (73.3)	215 (26.7)
Secondary school	4808 (46.0)	3443 (71.6)	1365 (28.4)
Undergraduate/bachelor’s degree	3120 (29.9)	2305 (73.9)	815 (26.1)
Master’s degree or higher	1721 (16.5)	1304 (75.8)	417 (24.2)

BMI, body mass index.

*BMI classification according to WHO criteria [35].

As summarized in Table 2, mean sleep duration and mean screen time were similar in the group of thin/normal weight children and the group of overweight/obese children. Only minor differences in the food consumption frequencies were observed between BMI categories. Comparing the five countries, Bulgaria, showing a higher-than-average prevalence of overweight (including obesity) (Table 1), had the lowest consumption frequencies of ‘*fresh fruits*’ and highest consumption frequencies of ‘*potato chips (crisps), corn chips, popcorn or peanuts*’ , ‘*candy bars or chocolate*’ , ‘*biscuits, cakes, doughnuts or pies*’ and ‘*pizza, French fries (chips), hamburgers*’ (Table 2). Also the mean screen time was higher in Bulgaria compared to the other countries (except Lithuania). However, Portugal being the country with the highest prevalence of overweight (including obesity) (Table 1) exhibited higher-than-average consumption frequencies of ‘*fresh fruit’* and lower-than-average consumption frequencies of ‘*potato chips (crisps), corn chips, popcorn or peanuts*’ , ‘*candy bars or chocolate*’ , ‘*biscuits, cakes, doughnuts or pies*’ and ‘*pizza, French fries (chips), hamburgers*’ (Table 2).

**Table 2:** **Means and standard deviations of exposure variables, covariates and outcome variables for the total study group and stratified by body mass index category and country**

**Variables under study**	**Total study group**	Thinness and normal weight*	Overweight (including obesity)*	**BUL** ^**†**^	**CZE** ^**†**^	**LTU** ^**†**^	**PRT** ^**†**^	**SWE** ^**†**^
	**Mean (SD)**							
**Exposure variables (hours/day)**								
Sleep duration	9.7 (0.9)	9.7 (0.9)	9.6 (0.9)	9.6 (1.1)	10.0 (0.8)	9.5 (0.8)	9.7 (0.8)	10.0 (0.7)
Screen time	2.2 (1.1)	2.2 (1.0)	2.3 (1.0)	2.5 (1.0)	1.7 (0.9)	2.6 (1.0)	1.8 (0.9)	1.9 (0.8)
TV time	1.5 (0.7)	1.5 (0.7)	1.6 (0.7)	1.8 (0.8)	1.2 (0.6)	1.8 (0.7)	1.3 (0.7)	1.3 (0.6)
PC time	0.7 (0.6)	0.7 (0.6)	0.7 (0.7)	0.7 (0.7)	0.5 (0.5)	0.9 (0.7)	0.5 (0.5)	0.6 (0.5)
**Covariates**								
Outdoor play time (hours/day)	2.0 (0.8)	2.0 (0.8)	2.0 (0.8)	2.2 (0.7)	2.1 (0.6)	2.2 (0.7)	1.4 (0.8)	2.1 (0.7)
BMI-for-Age z-score	0.36 (1.22)	-0.21 (0.78)	1.91 (0.76)	0.37 (1.36)	0.11 (1.20)	0.31 (1.19)	0.72 (1.21)	0.26 (1.06)
**Outcome variables – food consumption frequencies (days/week)**								
Fresh fruit	5.1 (2.1)	5.0 (2.1)	5.1 (2.1)	4.2 (2.1)	5.6 (1.8)	4.4 (2.1)	5.6 (2.0)	5.9 (1.8)
Vegetables (excluding potatoes)	4.6 (2.1)	4.6 (2.1)	4.5 (2.2)	4.2 (2.0)	4.5 (2.1)	4.0 (2.1)	4.7 (2.3)	5.6 (1.9)
100% fruit juice	2.8 (2.1)	2.8 (2.1)	2.7 (2.1)	3.2 (2.3)	2.0 (1.7)	3.1 (1.9)	2.5 (2.2)	2.5 (2.1)
Soft drinks containing sugar	2.5 (2.1)	2.5 (2.1)	2.5 (2.1)	3.1 (2.5)	3.6 (2.5)	2.4 (1.8)	2.1 (2.1)	2.0 (1.2)
Diet/light soft drinks	0.8 (1.3)	0.8 (1.3)	0.8 (1.4)	0.7 (1.4)	0.7 (1.5)	1.1 (1.2)	0.3 (1.0)	0.8 (1.3)
Low-fat/semi-skimmed milk	3.3 (2.9)	3.2 (2.9)	3.5 (3.0)	1.7 (2.0)	1.3 (2.2)	2.5 (2.5)	6.1 (2.1)	4.5 (2.8)
Whole-fat milk	2.2 (2.5)	2.3 (2.6)	1.9 (2.4)	2.8 (2.2)	2.6 (2.7)	3.0 (2.6)	0.7 (2.0)	1.5 (2.4)
Flavoured milk	1.7 (2.0)	1.7 (1.9)	1.8 (2.0)	1.2 (1.7)	1.8 (1.9)	1.5 (1.4)	3.0 (2.7)	1.6 (1.8)
Cheese	3.4 (2.2)	3.3 (2.2)	3.4 (2.2)	4.2 (2.1)	4.4 (2.1)	3.1 (2.0)	2.8 (2.3)	2.9 (2.2)
Yoghurt, cream cheese/quark or other dairy products	4.7 (2.1)	4.7 (2.2)	4.8 (2.1)	4.4 (2.1)	5.5 (1.8)	4.7 (2.1)	5.4 (2.0)	4.2 (2.3)
Meat	4.6 (1.9)	4.6 (1.9)	4.6 (1.9)	4.3 (2.1)	4.7 (1.7)	5.0 (1.9)	4.7 (1.9)	4.4 (1.6)
Fish	2.4 (1.5)	2.4 (1.4)	2.5 (1.5)	2.2 (1.4)	1.9 (1.0)	2.1 (1.2)	3.7 (1.9)	2.3 (1.1)
Potato chips (crisps), corn chips, popcorn or peanuts	2.2 (1.6)	2.2 (1.6)	2.1 (1.6)	3.6 (2.1)	1.6 (1.2)	2.1 (1.2)	1.5 (1.2)	1.7 (0.8)
Candy bars or chocolate	3.0 (1.9)	3.1 (1.9)	3.0 (1.9)	4.5 (2.2)	2.8 (1.7)	3.6 (2.0)	2.1 (1.4)	2.0 (0.6)
Biscuits, cakes, doughnuts or pies	2.8 (1.8)	2.9 (1.8)	2.8 (1.8)	3.6 (2.1)	2.7 (1.6)	3.1 (1.8)	2.7 (1.8)	2.0 (1.0)
Pizza, French fries (chips), hamburgers, sausages or meat pies	2.1 (1.4)	2.1 (1.4)	2.1 (1.4)	3.3 (2.0)	1.5 (1.1)	2.0 (1.1)	1.7 (1.1)	1.9 (0.7)

BMI, body mass index; SD, standard deviation.

*BMI classification according to the WHO criteria [35].

^**†**^The country codes refer to the International Organization for Standardization (ISO) 3166–1 Alpha-3 country codes: BUL: Bulgaria, CZE: Czech Republic, LTU: Lithuania, PRT: Portugal, SWE: Sweden.

Large differences in the food consumption frequencies were found between children with low versus high screen time and between children with short versus long sleep duration as is shown in Table 3. ‘*Fresh fruit*’ , ‘*vegetables (excluding potatoes)*’ , ‘*low-fat/semi-skimmed milk*’ and ‘*yoghurt, cream cheese/quark or other dairy products*’ consumption frequencies were higher in children with low screen time and in children with long sleep duration. The consumption frequencies of ‘*potato chips (crisps), corn chips, popcorn or peanuts*’ , ‘*candy bars or chocolate*’ , ‘*biscuits, cakes, doughnuts or pies*’ and ‘*pizza, French fries (chips), hamburgers*’ pointed to the opposite directions, i.e. were higher in children with high screen time as well as in children with short sleep duration.

**Table 3:** **Mean food consumption frequencies (days/week) by screen time and sleep duration category**

**Food items**	**Screen time**		**Sleep duration**	
	< 2 h/day*	≥ 2 h/day*	**< 9.7 h/day** ^**†**^	**≥ 9.7 h/day** ^**†**^
	**(n=4870)**	**(n=5583)**	**(n=4782)**	**(n=5671)**
	**Mean consumption frequencies in days per week (SD)**			
Fresh fruit	5.4 (2.0)	4.7 (2.2)	4.8 (2.2)	5.3 (2.1)
Vegetables (excluding potatoes)	4.9 (2.1)	4.3 (2.1)	4.4 (2.1)	4.8 (2.1)
100% fruit juice	2.6 (2.1)	2.9 (2.1)	2.7 (2.1)	2.8 (2.1)
Soft drinks containing sugar	2.3 (2.0)	2.8 (2.1)	2.5 (2.1)	2.5 (2.1)
Diet/light soft drinks	0.6 (1.2)	0.9 (1.4)	0.8 (1.3)	0.8 (1.3)
Low-fat/semi-skimmed milk	3.6 (3.0)	3.0 (2.8)	3.1 (2.9)	3.4 (3.0)
Whole-fat milk	2.0 (2.6)	2.4 (2.5)	2.2 (2.5)	2.2 (2.6)
Flavoured milk	1.8 (2.1)	1.7 (1.9)	1.7 (2.0)	1.7 (2.0)
Cheese	3.3 (2.3)	3.4 (2.2)	3.3 (2.2)	3.4 (2.3)
Yoghurt, cream cheese/quark or other dairy products	4.9 (2.2)	4.6 (2.1)	4.6 (2.1)	4.8 (2.2)
Meat	4.6 (1.8)	4.7 (1.9)	4.6 (1.9)	4.6 (1.9)
Fish	2.5 (1.5)	2.3 (1.4)	2.4 (1.4)	2.5 (1.5)
Potato chips (crisps), corn chips, popcorn or peanuts	1.8 (1.4)	2.5 (1.7)	2.2 (1.6)	2.1 (1.6)
Candy bars or chocolate	2.6 (1.6)	3.5 (2.1)	3.2 (2.0)	2.9 (1.9)
Biscuits, cakes, doughnuts or pies	2.6 (1.6)	3.1 (1.9)	2.9 (1.9)	2.8 (1.8)
Pizza, French fries (chips), hamburgers, sausages or meat pies	1.9 (1.2)	2.4 (1.5)	2.2 (1.5)	2.1 (1.4)

SD, standard deviation.

*This classification was based on the recommendation of the American Academy of Pediatrics to limit total media time to no more than 1 to 2 hours per day [32].

^**†**^This classification was based on the mean sleep duration of the total study population (9.7 hours/day).

The results of the multivariable analyses are presented in Table 4 (adjusted odds ratios (OR) and 99% confidence intervals (CI)). One additional hour of screen time was associated with higher consumption of ‘*100% fruit juice*’ (1.07 [1.01;1.14]; OR and 99% CI), ‘*soft drinks containing sugar*’ (1.28 [1.19;1.39]), ‘*diet/light soft drinks*’ (1.21 [1.14;1.29]), ‘*flavoured milk*’ (1.18 [1.08;1.28]), *‘meat’* (1.12 [1.05;1.19 ]), ‘*potato chips (crisps), corn chips, popcorn or peanuts*’ (1.32 [1.20;1.45]), ‘*candy bars or chocolate*’ (1.31 [1.22;1.40]), ‘*biscuits, cakes, doughnuts or pies*’ (1.22 [1.14;1.30]) and ‘*pizza, French fries (chips), hamburgers*’(1.30 [1.18;1.43]) and a lower consumption of ‘*fresh fruits’* (0.91 [0.86;0.97]) and ‘*vegetables (excluding potatoes)*’ (0.89 [0.83;0.95]). Sleep duration was found to be positively associated with consumption frequencies of ‘*fresh fruits*’ (1.11 [1.04;1.18]), ‘*vegetables (excluding potatoes)*’ (1.14 [1.07;1.23]), ‘*100% fruit juice*’ (1.14 [1.07;1.22]), ‘*whole-fat milk*’ (1.10 [1.03;1.18]), ‘*cheese*’ (1.13 [1.06;1.20]) and ‘*yoghurt, cream cheese/quark or other dairy products*’ (1.16 [1.08;1.24]). When additionally adjusting the models for BMI/A z-scores, odds ratios and confidence intervals remained almost unchanged. All associations pointed into the same directions and all significances persisted (see Additional file 1: Table S1).

**Table 4:** **Adjusted odds ratios** ^**†,#**^ **and 99%-confidence intervals for the effects of screen time (hours/day) and total sleep duration (hours/day) on consumption frequencies of selected food items**

**Outcome variables (Categorization: ≥ 4 days/week vs. < 4 days/week; exceptions in brackets)**	**Exposure variables**	
	**Screen time** ^**†**^ **(hours/day)**	**Sleep duration** ^**#**^ **(hours/day)**
	**OR (99% CI)**	**OR (99% CI)**
Fresh fruit (Daily vs. < 7 days/week)	0.91 (0.86;0.97)**	1.11 (1.04;1.18)**
Vegetables (excluding potatoes) (Daily vs. < 7 days/week)	0.89 (0.83;0.95)**	1.14 (1.07;1.23)**
100% fruit juice	1.07 (1.01;1.14)*	1.14 (1.06;1.22)**
Soft drinks containing sugar (≥ 1 day/week vs. never)	1.28 (1.19;1.39)**	0.99 (0.91;1.07)
Diet/light soft drinks (≥ 1 day/week vs. never)	1.21 (1.14;1.29)**	0.97 (0.90;1.04)
Low-fat/semi-skimmed milk	1.07 (1.00;1.15)	0.97 (0.90;1.05)
Whole-fat milk	0.97 (0.91;1.04)	1.10 (1.02;1.18)**
Flavoured milk	1.18 (1.08;1.28)**	0.98 (0.89;1.08)
Cheese	1.02 (0.97;1.09)	1.13 (1.06;1.20)**
Yoghurt, cream cheese/quark or other dairy products	0.95 (0.89;1.01)	1.16 (1.08;1.24)**
Meat	1.12 (1.05;1.19)**	1.04 (0.97;1.12)
Fish	0.95 (0.88;1.03)	1.06 (0.97;1.16)
Potato chips (crisps), corn chips, popcorn or peanuts	1.32 (1.20;1.45)**	1.07 (0.98;1.18)
Candy bars or chocolate	1.31 (1.22;1.40)**	1.03 (0.96;1.11)
Biscuits, cakes, doughnuts or pies	1.22 (1.14;1.30)**	1.03 (0.96;1.10)
Pizza, French fries (chips), hamburgers, sausages or meat pies	1.30 (1.18;1.43)**	1.03 (0.93;1.14)

CI, confidence interval; OR, odds ratio.

Significance levels: * p < 0.01 ** p < 0.001

^**†**^All models were adjusted for sleep duration, age, sex, outdoor play time and maximum parental educational level of the children and included random effects for country and primary sampling units.

^**#**^All models were adjusted for screen time, age, sex, outdoor play time and maximum parental educational level of the children and included random effects for country and primary sampling units.

In a sensitivity analysis, all models were run separately for either PC or TV time only instead of using the summary measure for total screen time to check whether results change when separating both screen behaviours (see Additional file 1: Table S2). Comparing the results for the summary measure with the results for the TV time only variable, all effect estimates pointed in the same directions, except for ‘*100% fruit juice*’ (no association found). The associations of PC time with each food item confirmed the results as found with the summary measure for screen time except for three food items where no associations were found (‘*fresh fruit*’. ‘*vegetables (excluding potatoes)*’ and ‘*meat*’).

## Discussion

Various studies analysed the effect of screen time or sleep duration on BMI status and put forth hypotheses regarding the underlying mechanisms [5,6,17,22,27,36,37]. Although suggested pathways include alterations in diet induced by reduced sleep duration or excessive screen time [24,36,38], most studies mainly investigate the effects of sleep duration or screen time on overweight/obesity but only few on food consumption [5-9,18-20,27], especially in young children. The present study provides evidence that both screen time and sleep duration independently affect children’s food frequency consumptions. Consistently with a recent review [14], screen time was found to be associated with consumption frequencies of energy-dense, micronutrient-poor foods which underlines the importance of limiting children’s screen time exposure. As the self-monitoring of foods consumed may be less pronounced when eating while doing something else, one explanation might be a passive overconsumption of foods high in saturated fats, free sugar or salt by children with high daily screen time exposure [37]. Moreover, food has been found to be the product category most frequently advertised on children’s TV [13]. A recent study on advertising in children reported that foods high in undesirable nutrients or energy were featured in 53 − 87% of the total food advertisements depending on the country under study [39]. Moreover, the rates of these food advertisements were found to be higher during children’s peak TV viewing times whereby diet drinks ranked among the highly advertised products [39]. This may explain the increased consumption of ‘*diet/light soft drinks’* in children with high screen time exposure that was observed in the present study. Beyond the potential effect of advertisement on food choice, several studies found associations between TV viewing and excess consumption of energy-dense foods [11,12], and commercials seem to promote overconsumption of the supplied snacks in children [15,38]. Children are exposed to advertisement not only while watching TV. A variety of online interactive techniques enables advertisers to reach young children and teenagers also when spending time in the Internet [40,41]. The lower fruit and vegetable consumption with increasing screen time found in our study may reflect a substitution effect resulting from the accessibility of healthy/unhealthy alternatives as well as from requests for unhealthy foods triggered by other factors notably advertisements. In a literature review done by Rasmussen et al., availability of unhealthy competitive food options was reported to be a barrier to eating fruits and vegetables in children [42]. However, due to the lack of a direct measure of advertisement in this study, the real effect of advertisement on children’s food choices cannot be evaluated. In particular, further research on the role of new media technologies in exacerbating exposure to advertisements or encouraging sedentary behaviour is needed [43].

The analysis on the second exposure variable addressed in this study revealed that an additional hour of sleep was positively associated mainly with consumption frequencies of ‘*fresh fruits*’ , ‘*vegetables (excluding potatoes)*’ , ‘*100% fruit juice*’ , ‘*cheese*’ and ‘*yoghurt, cream cheese/quark or other dairy products’*. Different mechanisms may explain this finding. Children with long sleep duration might be those watching less TV and, hence, those being less exposed to advertisement. In a recent study by Chahal et al. conducted in fifth grade Canadian students, access to and night-time use of electronic media were reported to be associated with shortened sleep duration, excess body weight, poorer diet quality and lower physical activity levels [44]. However, in our analysis no evidence for a mediation effect was found, i.e. the effect of screen time seems to act independently from the effect of sleep duration on food consumption. Another explanation might be neurohormonal changes in periods of reduced sleep that were shown to increase appetite and, consequently, presumably increase caloric intake [19,23,24,45]. The exact mechanisms leading to the differences in food choices between children with short versus long sleep durations cannot be determined based on our cross-sectional data analyses and remain an area for future studies.

In a second analysis step, BMI/A z-score was added as potential confounder variable to all models. Weight status is known to be associated with sleep duration and screen time, but also with food consumption. However, the directions of the three listed associations are unclear yet making it difficult to decide whether weight status is indeed a confounder in the associations under investigation. For instance, there is evidence that obesity affects sleep, but also that sleep patterns are related to weight [46]. All effect estimates remained almost unchanged when including BMI/A z-score in the models, such that considering BMI/A z-score as a confounder did not alter our results.

Parenting practices are likely to differ among educational levels of parents and may significantly affect children’s leisure time behaviours [47]. In our study, strong associations were also observed between the maximum educational level of parents and food consumption frequencies of the children. A high educational level of the parents (bachelor’s/master’s degree or higher) was found to be negatively associated with the consumption of foods high in fat, free sugar or salt (‘*soft drinks containing sugar*’ , ‘*flavoured milk*’ , ‘*potato chips (crisps), corn chips, popcorn or peanuts*’ , ‘*candy bars or chocolate*’ , ‘*biscuits, cakes, doughnuts or pies*’ , ‘*pizza, French fries (chips), hamburgers*’) and positively associated with the consumption frequencies of ‘*fresh fruits*’ and ‘*vegetables (excluding potatoes)*’ indicating a need to target, in particular, parents with lower educational levels in intervention programmes. Apart from the provision of individualized guidance to parents regarding children’s sleep duration and screen time, the design of new policies on food marketing communications to children, or strengthening of existing ones, could be effective population-based approaches [48].

### Limitations and strengths

Because COSI has a cross-sectional design its results can only provide indications of associations but cannot make causal inferences. For example, sleep may not only affect diet; there is also certain evidence that diet affects sleep duration and quality [49]. Further limitations include the parentally reported sleep duration and screen time variables. As parents may usually observe the bed and get-up times of their children rather than the times the child is falling asleep or waking up, this may have introduced some error. Nevertheless, as this error is likely to be random, it might have attenuated the observed associations but is unlikely to bias the effect estimates. Also the parental estimates of their children’s screen time may be error-prone, especially if the child has media devices in the bedroom or spends a lot of time out of home. No information on media in the child’s bedroom was available, which would have been an important covariate to include.

Measurement error in dietary intake data is one of the largest challenges in nutritional epidemiology. In this study, misreporting cannot be precluded, in particular underreporting of specific food items by parents with overweight or obese children may have occurred [50]. Apart from this, the COSI food frequency consumption list was designed as an easily applicable monitoring tool to get an overall indication of the children’s usual consumption frequencies of a food group (e.g. “fish”) but was not designed to capture difference in consumption of the “healthier” and “less healthier” options within each food group (e.g. “fish fingers” versus “broiled low-fat fish”) and did not include portion sizes. The main strength of this study is the simultaneous investigation of two risk factors for less and more favourable food choices, which enabled us to consider and also interpret their potential interrelations. Further strengths include the large sample size of more than 10 000 children, the standardized assessment procedures of COSI and country-based sampling strategies designed to yield nationally representative data [29].

## Conclusion

The results suggest a potential relation between high screen time exposure and increased consumption frequencies of foods high in fat, free sugar or salt whereas long sleep duration may favourably be related to children’s food choices. Our results provide useful suggestions for the design of overweight intervention programmes aiming to modify sleep and screen time behaviours and address the frequency consumption of particular food items.

## Additional file

Additional file 1: Table S1. Adjusted odds ratios^†,#^ and 99%-confidence intervals for the effects of screen time (hours/day) and total sleep duration (hours/day) on consumption frequencies of selected food items; models additionally adjusted for BMI-for-age z-scores of the children. **Table S2.** Results of the sensitivity analysis: Adjusted odds ratios^†^ and 99%- confidence intervals for the effects of TV time (hours/day) and PC time (hours/day) on consumption frequencies of selected food items.

## Abbreviations

BMI: Body mass index
BMI/A: Body mass index-for-age
CI: Confidence interval
COSI: Childhood Obesity Surveillance Initiative
OR: Odds ratio
PC: Personal computer
SD: Standard deviation
TV: Television
WHO: World Health Organization
