# Supplementary material for: WHO European Childhood Obesity Surveillance Initiative: associations between sleep duration, screen time and food consumption frequencies
Source: BMC Public Health. 2015 Apr 30;15:442. doi: 10.1186/s12889-015-1793-3 (PMC4440513; doi:10.1186/s12889-015-1793-3)
Supplement: Additional file 1: Table S1. — Adjusted odds ratios†,# and 99%-confidence intervals for the effects of screen time (hours/day) and total sleep duration (hours/day) on consumption frequencies of selected food items; models additionally adjusted for BMI-for-age z-scores of the children. Table S2. Results of the sensitivity analysis: Adjusted odds ratios† and 99%- confidence intervals for the effects of TV time (hours/day) and PC time (hours/day) on consumption frequencies of selected food items. [file 12889_2015_1793_MOESM1_ESM.docx]

**WHO European Childhood Obesity Surveillance Initiative:** **associations between sleep duration, screen time and food consumption frequencies**

Claudia Börnhorst, Trudy MA Wijnhoven, Marie Kunešová, Agneta Yngve, Ana I Rito, Lauren Lissner, Vesselka Duleva, Ausra Petrauskiene, João Breda

© 2015 World Health Organization; licensee BioMed Central Ltd.

**Supplementary file S1 - Adjusted odds ratios^†,#^ and 99%-confidence intervals for the effects of screen time (hours/day) and total sleep duration (hours/day) on consumption frequencies of selected food items; models additionally adjusted for BMI-for-age z-scores of the children**

|  | **Exposure variables** | |
| --- | --- | --- |
|  | **Screen time^†^ (hours/day)** | **Sleep duration^#^ (hours/day)** |
| **Outcome variables** (Categorization: ≥ 4 days/week vs. < 4 days/week; exceptions in brackets) | **OR (99% CI)** | |
| Fresh fruit (Daily vs. < 7 days/week) | 0.91 (0.85;0.97)** | 1.11 (1.04;1.19)** |
| Vegetables (excluding potatoes) (Daily vs. < 7 days/week) | 0.89 (0.83;0.95)** | 1.14 (1.07;1.23)** |
| 100% fruit juice | 1.08 (1.01;1.15)* | 1.13 (1.06;1.21)** |
| Soft drinks containing sugar (≥ 1 day/week vs. never) | 1.29 (1.19;1.39)** | 0.99 (0.91;1.07) |
| Diet/light soft drinks (≥ 1 day/week vs. never) | 1.20 (1.13;1.28)** | 0.98 (0.91;1.05) |
| Low-fat/semi-skimmed milk | 1.07 (1.00;1.14) | 0.97 (0.90;1.05) |
| Whole-fat milk | 0.98 (0.92;1.05) | 1.09 (1.02;1.17)** |
| Flavoured milk | 1.18 (1.09;1.29)** | 0.98 (0.89;1.07) |
| Cheese | 1.02 (0.96;1.08) | 1.13 (1.06;1.21)** |
| Yoghurt, cream cheese/quark or other dairy products | 0.95 (0.89;1.01) | 1.16 (1.08;1.24)** |
| Meat | 1.12 (1.05;1.19)** | 1.04 (0.97;1.12) |
| Fish | 0.96 (0.88;1.04) | 1.06 (0.97;1.16) |
| Potato chips (crisps), corn chips, popcorn or peanuts | 1.33 (1.21;1.46)** | 1.07 (0.97;1.18) |
| Candy bars or chocolate | 1.32 (1.23;1.41)** | 1.03 (0.96;1.11) |
| Biscuits, cakes, doughnuts or pies | 1.23 (1.15;1.31)** | 1.02 (0.96;1.10) |
| Pizza, French fries (chips), hamburgers, sausages or meat pies | 1.31 (1.19;1.44)** | 1.03 (0.93;1.13) |

CI, confidence interval; OR, odds ratio.

Significance levels: * p < 0.01 ** p < 0.001

**^†^**All models were adjusted for sleep duration, age, sex, BMI-for-age z-scores of the children, outdoor play time and maximum parental educational level of the children and included random effects for country and primary sampling units.

**^#^**All models were adjusted for screen time, age, sex, BMI-for-age z-scores of the children, outdoor play time and maximum parental educational level of the children and included random effects for country and primary sampling units.

**Supplementary file S2 - Results of the sensitivity analysis: Adjusted odds ratios^†^ and 99%- confidence intervals for the effects of TV time (hours/day) and PC time (hours/day) on consumption frequencies of selected food items**

|  | **Exposure variables** | |
| --- | --- | --- |
|  | **TV time^†^ (hours/day)** | **PC time^†^ (hours/day)** |
| **Outcome variables** (Categorization: ≥ 4 days/week vs. < 4 days/week; exceptions in brackets) | **OR (99% CI)** | |
| Fresh fruit (Daily vs. < 7 days/week) | 0.86 (0.79;0.94)** | 0.97 (0.88;1.07) |
| Vegetables (excluding potatoes) (Daily vs. < 7 days/week) | 0.81 (0.74;0.89)** | 0.97 (0.88;1.08) |
| 100% fruit juice | 1.00 (0.91;1.09) | 1.18 (1.08;1.30)** |
| Soft drinks containing sugar (≥ 1 day/week vs. never) | 1.34 (1.21;1.49)** | 1.25 (1.11;1.41)** |
| Diet/light soft drinks (≥ 1 day/week vs. never) | 1.21 (1.10;1.32)** | 1.24 (1.13;1.36)** |
| Low-fat/semi-skimmed milk | 1.11 (1.01;1.22)* | 1.05 (0.94;1.16) |
| Whole-fat milk | 0.97 (0.88;1.06) | 0.98 (0.88;1.08) |
| Flavoured milk | 1.20 (1.07;1.35)** | 1.18 (1.03;1.36)** |
| Cheese | 0.99 (0.91;1.08) | 1.07 (0.97;1.17) |
| Yoghurt, cream cheese/quark or other dairy products | 0.94 (0.87;1.03) | 0.95 (0.87;1.05) |
| Meat | 1.15 (1.05;1.25)** | 1.10 (1.00;1.22) |
| Fish | 0.91 (0.81;1.02) | 1.00 (0.88;1.14) |
| Potato chips (crisps), corn chips, popcorn or peanuts | 1.41 (1.24;1.61)** | 1.24 (1.08;1.42)** |
| Candy bars or chocolate | 1.38 (1.25;1.52)** | 1.26 (1.14;1.39)** |
| Biscuits, cakes, doughnuts or pies | 1.32 (1.21;1.45)** | 1.14 (1.04;1.26)** |
| Pizza, French fries (chips), hamburgers, sausages or meat pies | 1.37 (1.20;1.57)** | 1.26 (1.09;1.45)** |

CI, confidence interval; OR, odds ratio.

Significance levels: * p < 0.01 ** p < 0.001

**^†^**All models were adjusted for sleep duration, age, sex, outdoor play time and maximum parental educational level of the children and included random effects for country and primary sampling units.
